# *Haplometra cylindracea* (Zeder, 1800) (Trematoda: Plagiorchiidae): variation in the dates of cercarial shedding for overwintering *Galba truncatula*

**DOI:** 10.1051/parasite/2011182181

**Published:** 2011-05-15

**Authors:** P. Vignoles, G. Dreyfuss, D. Rondelaud

**Affiliations:** 1 UPRES EA no. 3174, Faculties of Medicine and Pharmacy 87025 Limoges France

**Keywords:** cercaria, cercarial shedding, *Galba truncatula*, *Haplometra cylindracea*, natural infection, cercaire, émission cercarienne, *Galba truncatula*, *Haplometra cylindracea*, infestation naturelle

## Abstract

Natural infections of *Galba truncatula* with *Haplometra cylindracea* were followed from 2001 to 2009 to determine if their characteristics were similar when snails came from water collections frequented by *Bufo bufo* or by frogs and newts for their egg-laying. Snail samples were collected from both types of sites to count shed cercariae for three days and also free cercariae when snails were dissected. In sites only frequented by *B. bufo*, cercarial shedding occurred earlier than in those colonized by frogs and newts (March instead of April-May). In contrast, the number of cercariae shed during three successive days was significantly higher in May. This variation in the dates of cercarial shedding might be due, either to a synchronism between cercaria-releasing snails and the presence of the definitive host (tadpoles) in water collections, or to an earlier infection of overwintering snails in autumn by *H. cylindracea* miracidia in the case of toad-frequented sites.

## Abstract (fr)

L’infestation naturelle de *Galba truncatula* par *Haplometra cylindracea* a été suivie de 2001 à 2009 pour déterminer si les caractéristiques du parasitisme sont identiques lorsque les mollusques proviennent de collections d’eau fréquentées par *Bufo bufo* pour ses pontes ou de celles occupées uniquement par des grenouilles et des tritons. Des échantillons de limnées ont été récoltés dans les deux types de sites pour compter les cercaires émises pendant trois jours et aussi celles qui sont libres dans le corps des mollusques après leur dissection. Dans les habitats fréquentés seulement par *B. bufo*, les émissions cercariennes se produisent plus tôt que dans les sites colonisés par les grenouilles et les tritons (en mars au lieu d’avril-mai). Par contre, le nombre de cercaires émises pendant les trois jours successifs est significativement plus élevé en mai. Cette variation dans les dates des émissions cercariennes peut être due soit à une synchronisation entre l’émission des larves par les mollusques et la présence de l’hôte définitif (têtards) dans les collections d’eau, soit à une infestation plus précoce des mollusques transhivernants par les miracidiums de *H. cylindracea* au cours de l’automne dans les sites fréquentés par *B. bufo*.

In the department of Haute-Vienne (central France), the main intermediate host of *Haplometra cylindracea* (lung fluke of amphibians) is *Galba truncatula*. According to Rondelaud *et al.* (2009), the prevalence of natural infections in this snail may be high (up to 78%). However, natural fluctuations of this infection rate according to snail habitat, the year of study, and also the month of the year were reported by Hourdin *et al.* (1991) and Goumghar *et al.* (2000). In swampy meadows in Haute-Vienne, cercarial shedding of *H. cylindracea* occurred in April-May and September-October as those of *Fasciola hepatica* and *Paramphistomum daubneyi* (Rondelaud *et al.*, 2009). In contrast, in road ditches in the same region, the dates of shedding in spring were more variable, either at mid-March, or at the end of April-beginning of May. In view of this variability, the following question arose: was this variability caused by seasonal variations in the microclimate of *G. truncatula* habitats? Or did it result from the behaviour of the amphibian species infected by *H. cylindracea*? To verify either possibility, field investigations on snail populations were carried out since 2000 in northern Haute-Vienne in different water collections located in road ditches and frequented by different amphibian species.

Among the 77 sites followed by our team from the 2000 s, seven dirt tracks and road ditches were chosen by *Bufo bufo* for its egg-laying and were never frequented by frogs and newts because of early drying of these habitats (generally at the end of April). 11 others were only colonized by frogs (*Rana dalmatina*, *Rana esculenta*) and newts (*Triturus helveticus*), while the 59 others were frequented by different species of frogs, toads and/or newts. A road ditch (46° 66’ 56” N, 1° 10’ 57” E) located on the commune of Rancon (site *i*) and a dirt track (46° 52’ 51” N, 1° 5’ 50” E) on that of Veyrac (site *ii*), only frequented by *B. bufo*, were followed from 2001 to 2009. Two other ditches only frequented by frogs and newts were also considered: the first (45° 56’ 7” N, 1° 11’ 8” E) is located on the commune of Nieul (site *iii*), whereas the other ditch (46° 7’ 42” N, 1° 11’ 2” E) is situated on the commune of Rancon (site *iv*). These four stations were chosen because of small distances between sites *i* and *iv* (500 m), or between sites *ii* and *iii* (10.5 km). In each habitat, a population of *G. truncatula* was living.

When a natural infection with *H. cylindracea* in snails was found on November or December in either site, monthly samples of 8-15 adult snails each were collected the next year from mid-January to mid-May. The low number of *G. truncatula* collected from each site for each sampling date was chosen because of the small size of snail populations in these ditches (< 100 overwintering snails per site) and the high prevalence of infection (often > 50%) found in *H. cylindracea*infected snails on acid soil (Rondelaud *et al.*, 2009). When sites were watered, temperature was measured at 2 p.m. in the most superficial layer (thickness, 4 cm) of water. Snails were first subjected to an artificial light for three days at a constant temperature of 20 °C to stimulate cercarial shedding and count shed cercariae. They were then dissected under a stereomicroscope to count free cercariae within each snail. Lastly, samples of 20 (2007) or 30 (2009) cercariae each were randomly collected from cercariae-shedding snails originating from three sites. Cercariae were heat killed and several lengths were measured with an ocular micrometer according to the parameters used by Combes (1968) and Grabda-Kazubska (1970) for the study of plagiorchiid cercariae. These measurements were performed to analyze the morphological variability of *H. cylindracea* cercariae according to *G. truncatula* populations and also to determine if a close related plagiorchiid species: *Haplometra brevicaeca*, known to infect toads (Timon-David, 1961), would not be present in snail populations living in ditches frequented by *B. bufo* (sites *i* and *ii*).

The first three parameters were the prevalence of snail infection for each site and each year, the number of shed cercariae, and that of free cercariae present in the body of snails after their dissection. Measurements performed on heat-killed cercariae were the total length of the body, that of the tail, the diameter of both suckers, and the length of the stylet. Water temperature was also considered. Individual values recorded for each parameter and each type of site (stations only frequented by *B. bufo*, or by frogs and newts) between 2001 and 2009 were pooled and were expressed for a single year, taking into account sampling month. A χ^2^ test and one-way analysis of variance (Stat-Itcf, 1988) were used to determine levels of significance.

In sites only frequented by *B. bufo*, the temperature of water at 2 p.m. was 5.3 °C (± 0.9 °C) at mid-January and increased up to 14.1 °C (± 1.3 °C) at mid-April (data not shown). Similar findings were also noted in the other two ditches, with values ranging from 5.8 °C ± 1.0 °C (at mid-January) to 15.4 °C ± 2.6 °C (at mid-May). The differences between both types of sites were not significant, whatever the date of snail sampling.

The annual prevalence of *H. cylindracea* infection (Table I) in snails ranged from 28.5% to 68.0% in sites only frequented by *B. bufo* and from 23.4% to 63.0% in the other two ditches. If the overall prevalence for each site was considered, the percentages ranged from 38.5% to 53.0% and no significant difference was noted. Table II lists the numbers of snails shedding cercariae and the quantities of these larvae for each sampling month. In sites only frequented by *B. bufo*, snails released their cercariae from February to April with a peak in March. Another peak in the number of shed cercariae was also noted in March. In contrast, cercarial shedding occurred later in sites frequented by frogs and newts. The highest number of snails and that of shed cercariae were noted in May. The difference between the numbers of cercariae shed in March (sites with *B. bufo*) and May (sites with frogs and newts) was significant (*F* = 4.25, *P* < 0.05). The presence of free cercariae in dissected snails was noted from February to April in toad-frequented sites and from March to May in the other ditches. The highest number of these cercariae was noted in March in the former sites and in May in the latter but the difference was not significant.

**Table 1.** Total number of *G. truncatula* collected from each site and overall prevalence of *H. cylindracea* infection between 2001 and 2009.

Snail site		Year	Total number of snails collected	Number of snails infected with *H. cylindracea* (prevalence in %)	Overall prevalence of *H. cylindracea* infection (%)
Sites only frequented by *B. bufo*	Rancon (site i)	2004	33	11 (33.3)	38.5
		2005	37	16 (43.2)	
	Veyrac (site ii)	2001	55	24 (43.6)	43.0
		2002	51	17 (33.3)	
		2005	47	32 (68.0)	
		2007	44	19 (43.1)	
		2009	49	14 (28.5)	

Sites frequented by frogs and newts	Nieul (site iii)	2002	54	34 (62.9)	45.1
		2004	47	11 (23.4)	
		2009	34	16 (47.0)	
	Rancon (site iv)	2004	49	26 (53.0)	50.3
		2006	54	20 (37.0)	
		2007	46	29 (63.0)

**Table 2.** Mean numbers (S.D.) of *H. cylindreacea* cercariae shed by infected snails or counted in snails after their dissection. Individual values recorded for each parameter between 2001 and 2009 were pooled and were expressed for a single year.

			Shed cercariae		Free cercariae	
	Date of snail sampling	Number of infected snails	Number of snails	Number of cercariae in three days	Number of dissected snails	Number of cercariae
Sites *i* and *ii* only frequented by *B. bufo*	January	33	0	-	17	15.1 (9.2)
	February	39	7	87.2 (37.8)	29	27.4 (16.9)
	March	43	41	215.3 (125.0)	37	55.3 (29.0)
	April	17	15	113.5 (82.7)	17	15.6 (11.5)
Sites *iii* and *iv* frequented by frogs and newts	January	19	0	-	0	-
	February	26	0	-	4	3.1 (2.4)
	March	22	2	59.8 (32.1)	13	11.4 (8.1)
	April	38	23	234.6 (119.4)	35	25.3 (14.0)
	May	31	30	457.1 (219.0)	30	43.5 (21.4)

Table III gives the values of several parameters for cercariae shed by snails originating from sites *ii*, *iii* and *iv*. No significant difference between the mean lengths of cercarial bodies was noted. Similar findings were also found for the length of the tail, the diameter of the oral sucker, that of the ventral sucker, and the length of the stylet. All cercariae showed the typical morphology of *H. cylindracea*.

**Table 3.** Mean values (S.D.) of five parameters measured on some heat-killed cercariae of *H. cylindracea* after their shedding from snails originating from three sites.

Parameters	Site *ii* (2007 + 2009)	Site *iii* (2007)	Site *iv* (2009)
Number of cercariae	20 + 30	20	30
Total length of the body (μm)	406.4 (19.6)	395.7 (15.8)	402.7 (13.2)
Total length of the tail (μm)	296.1 (23.9)	283.5 (19.8)	287.3 (21.2)
Diameter of the oral sucker (μm)	73.2 (2.5)	71.3 (2.9)	71.0 (1.7)
Diameter of the ventral sucker (μm)	68.4 (2.7)	66.2 (1.8)	67.4 (1.4)
Length of the stylet (μm)	30.7 (0.4)	30.7 (0.5)	31.0 (0.8)

This variability in the dates of cercarial shedding according to the geographical location of snails is difficult to explain because the larval development of a digenean within its snail host is closely related to temperature (Smyth & Halton, 1983). The role of temperature cannot be here held to explain this finding owing to insignificant differences between temperatures measured in the four sites. In the same way, the presence of another plagiorchiid species in sites only frequented by toads cannot be proposed because the lengths and diameters measured on *H. cylindracea* cercariae (Table III) did not significantly differ from each other. Two perhaps complementary hypotheses might explain the variability noted for the dates of cercarial shedding. The first is to admit that *H. cylindracea* cercariae would be released during the period where tadpoles are present in water collections (in March for those of *B. bufo*, in May for those of frogs). If this assumption is valid, it would indicate the existence of a synchronism between cercarial shedding and the presence of the definitive host, as that reported for several schistosome species (see review by Combes, 1995). The second hypothesis concerns the infection of overwintering snails by *H. cylindracea* miracidia: in toad-frequented sites, the infection of snails would occur earlier than that happening in ditches frequented by frogs and newts so that cercarial shedding took place in March in the former sites in spite of winter conditions.

The other findings reported in the present study warrant special comments. First, the numbers of shed cercariae disagreed with those reported by Moukrim *et al.* (1993) for other *G. truncatula* naturally infected with *H. cylindracea* on acid soil. According to these authors, each snail released 500-600 cercariae per day (at 20 °C), whereas 215 cercariae in March and 457 in May (Table II) exited from snails during three days. In our opinion, the most valid hypothesis is to admit that larval development of *H. cylindracea* would be less productive in overwintering snails (the present study) than in those belonging to spring generation (the report by Moukrim *et al.*, 1993). Secondly, the number of free cercariae (Table II) was enough high in the body of snails, whatever the type of site, whereas dissection was performed after three successive days of cercarial shedding. As these *G. truncatula* were subjected in the laboratory to a constant temperature greater than that existing in their natural habitat, the important release of the most differentiated cercariae would have caused the exit of immature cercariae from their parental sporocysts and this exit would be greater than usually. Lastly, even if the different lengths recorded for *H. cylindracea* cercariae (Table III) ranged in the scale of values reported by Combes (1968) and Vala (1973), the slight differences noted between the three sites for each parameter suggest interpopulation variability in the morphology of these cercariae and this finding confirms the polymorphism already reported by Grabda-Kazubska & Combes (1981) for this digenean.

## References

[R1] Combes C. Biologie, écologie des cycles et biogéographie de Digènes et Monogènes d’Amphibiens dans les Pyrénées. Mémoires du Muséum National d’Histoire Naturelle, 1968, 51, 1–191

[R2] Combes C. Interactions durables. Écologie et évolution du parasitisme. Masson, Paris, 1995, 544 p.

[R3] Goumghar M.D., Abrous M., Ferdonnet D., Dreyfuss G. & Rondelaud D.. Prevalence of *Haplometra cylindracea* infection in three species of *Lymnaea* snails in central France. Parasitology Research, 2000, 86, 337–339 10780746 10.1007/s004360050054

[R4] Grabda-Kazubska B. Studies on the life cycle of *Haplometra cylindracea* (Zeder, 1800) (Trematoda: Plagiorchiidae). Acta Parasitologica Polonica, 1970, 18, 497–512

[R5] Grabda-Kazubska B. & Combes C. Morphological variability of *Haplometra cylindracea* (Zeder, 1800) (Trematoda: Plagiorchiidae). Acta Parasitologica Polonica, 1981, 28, 39–63

[R6] Hourdin D., Moukrim A. & Rondelaud D. L’infestation naturelle de *Lymnaea truncatula* Müller par *Haplometra cylindracea* Zeder et *Notocotylus* sp. À propos de quelques observations de terrain. Revue de Médecine Vétérinaire de Toulouse, 1991, 142, 139–142

[R7] Moukrim A., Oviedo J., Vareille-Morel C., Rondelaud D. & Mas-Coma S. Cercarial sheddings of *Haplometra cylindracea.* About several observations in *Lymnaea truncatula* during single or dual infections. Research and Reviews in Parasitology, 1993, 53, 57–61

[R8] Rondelaud D., Vignoles P. & Dreyfuss G. La Limnée tronquée, un mollusque d’intérêt médical et vétérinaire. Pulim, Limoges, 2009, 283 p.

[R9] Smyth J.D. & Halton D.W. Physiology of trematodes. 2d edition. Cambridge University Press, Cambridge, 1983, 446 pp.

[R10] Stat-Itcf Manuel d’utilisation. Institut technique des céréales et des fourrages, Service des études statistiques, Boigneville, 1988, 210 pp.

[R11] Timon-David J. Note préliminaire sur la distomatose pulmonaire du crapaud en Provence. 86ème Congrès des Sociétés Savantes, Montpellier, 1961, 683–687

[R12] Vala J.C. Étude écologique du parasitisme des mollusques de la Mosson, hôtes intermédiaires de Trématodes. Thèse de doctorat, Université de Montpellier, Montpellier, 1973, no. 1455, 174 p.

